# Further detail required to assess additional evidence on agreement between VITEK 2 and broth microdilution piperacillin/tazobactam testing in cephalosporin resistant Enterobacterales

**DOI:** 10.1128/jcm.00293-26

**Published:** 2026-05-14

**Authors:** Andrew J. McArdle, Victoria Austin

**Affiliations:** 1 Alder Hey Children's Hospital 200462, Liverpool, United Kingdom; Mayo Clinic, Rochester, Minnesota, USA

**Keywords:** AMR, Vitek

## LETTER

We read with interest the study by Richter et al. evaluating VITEK 2 and MicroScan for testing of ceftriaxone non-susceptible Enterobacterales (1). Given the previous issue with piperacillin/tazobactam (PT) testing using VITEK 2 (2), and more recent concerns about agreement between multiple methodologies and broth microdilution (BMD) arising from the MERINO-2 trial, this issue is very relevant.

This study provides results that are supportive of VITEK 2’s accuracy in reporting PT susceptibility, with no very major errors (VME) among 56 isolates resistant by formal MIC testing. This is noted to be discrepant with some other studies, with the smaller size of these studies offered as a potential explanation.

For example, Sohani et al. reported a VME rate of 4/15, with a reported MIC of 8 by VITEK 2 accounting for the errors (3). Manuel et al. report a VME rate of 1/16.(4)

Henderson et al. assessed only isolates classified as PT susceptible by routine laboratory testing (mostly VITEK 2); therefore, a VME rate is not calculable (5). However, they found that 18/319 (6%) were resistant by BMD. Of 298 isolates that were again reported as PT susceptible by VITEK 2, 12 were resistant by BMD (4%). Misclassifications were noted with VITEK 2 MIC ≤ 4 (6/188 = 3%) and MIC = 8 (6/79 = 8%). MERINO excluded 250 patients screened for inclusion due to isolates being PT non-susceptible, with approximately half of eligible patients being recruited into the study. With 84% of isolates selected for the study, this suggests that there would be around 104 isolates reported as PT resistant from consecutive isolates to correspond to the 319 included in the study. A ballpark VME estimate might therefore be 12/116 (~10%).

Sohani et al. studied consecutive bloodstream isolates (3). Manuel et al. selected isolates from a single year to represent the species and PT MIC distributions of the SENTRY study (4). Henderson et al. provided isolates from patients recruited into the MERINO study (5).

We are concerned that the study report from Richter et al. does not give any details of how the 200 isolates were selected from the 5-year SENTRY program and CDC bank (1). While the SENTRY program receives consecutive isolates from participating laboratories (up to a monthly limit per laboratory) (6), the CDC bank is built from organisms under surveillance, outbreak investigations, and submissions for reference testing (7). The isolates may represent a subset of the 248 in Carvalhaes et al. that were resistant to ceftriaxone by BMD (8).

We consider that detail on the characteristics of eligible isolates that were selected for the study and those that were not is vital to assess the risk of spectrum bias. For example, omission by any means of isolates with borderline MICs (or favorable inclusion of isolates with extreme MICs) would artificially inflate agreement. Further to this, we invite the authors to provide a full presentation of the comparative MIC data as in Sohani et al. and Henderson et al. (3, 4).
